# Account of the Effects of Camphor in a Case of Insanity

**Published:** 1785

**Authors:** 


III. *Account of the Effects of Camphor in a Caſe of Inſanity. By* William Oliver, M. D. *Phyſician Extraordinary to his Royal Highneſs the Prince of Wales.*

FEBRUARY 23d, 1781, I was called to ſee a gentleman who ſome few days before had been ſeized with mania; he continued in this ſtate, with very few intervals of reaſon, until the month of October. During this ſtate, the uſual means were employed for his recovery, very, and these means were sanctified, by the attendance of Dr. Monro.

Early in the month of October, I proposed making a trial of Camphor: Dr. Monro seemed to have no opinion of the medicine, nor had the late Dr. Battie, as I have heard, from good authority; I prevailed however, and a dose of ʒij was given, in a draught, on the 16th of October, about mid-day.

The patient was at this time in a more tranquil state than he had been for most part of the time before; for now, he walked about his garden, played at skettles with his keepers, and was seldom so outrageous as to need binding.

Upon visiting my patient next day, I found that in a quarter of an hour after the draught was taken, he fell down on the pavement, adjoining to the skettle-alley, where the medicine was given.

The two keepers, and his own servant, who were present, described him, as having turned sick and pale, before his fall; they took him up, carried him to his bed, and in a little time he came to himself again.

The patient had all along his speech affected, and many paralytic symptoms preceded the mania.

The Camphor was not repeated; 'twas offered, but he would not take it. Now, from this day, he became more rational, and viſibly altered for the better; and he now went out airing in a coach every day; put on his uſual dreſs, and behaviour, and on the 30th of October he told us, that in the preceding night, whilſt he was in bed, his ſenſes returned to him, and ſomething like a flaſh of lightning, he ſaid, preceded their return. He now quitted his confinement, and trials were made of his behaviour in various companies, at different houſes. Parties were formed for him, at his own houſe; he became natural, eaſy, polite, and in every reſpect like himſelf, and played his game at whiſt, with great accuracy, moſt evenings. One keeper was diſmiſſed, and the other kept out of ſight. Thus we endeavoured to amuſe him, and improve and ſtrengthen his returning ſenſes, by proper company, till the 21ſt of November, when he ſet out, with a near relation of his, for the country, where he ſat down in a ſober, quiet family, rejoicing at his recovery, and poſſeſſed of the ſtrongeſt reſolutions to lead an abſtemious life, and to join with it conſtant exerciſe; all which he moſt exactly obſerved, and ſoon recovered his ſtrength and fleſh, and even grew fat.

In February, 1783, buſineſs called him to London, and he attended Parliament; I ſaw him on his arrival, and frequently viſited him; he was perfectly well.

On the 9th of the ſucceeding April, he complained of ſickneſs at his ſtomach, grew triſt, deſponding, and a true hypochondria was formed; which ſoon grew into the higheſt degree of melancholia. He continued in this ſtate through the whole ſummer; during the months of July, Auguſt, and September, he was fed by his ſervant in general, and all his excretions ſeemed involuntary.

It now occurred to me, that the fit ariſing from the doſe of Camphor, given in the year 1781, might have been the means of his recovery at that time; and now, as I had nobody to conſult with, I reſolved on trying it again, in the doſe of ℈ij, as mentioned by Hoffman, and which caſe, as recited by him, I was unacquainted with in the year 1781.

Accordingly, on the 15th of October, 1783, I gave it to him, from my own hand; and in about ten minutes, he reeled, and fell into my arms; I placed him in an elbow chair, where he turned ſick, and retched, but nothing came up; he now grew pale, trembled all over, his eyes cloſed, his head drooped, and thick rheum ran from his mouth. His pulſe was weak, intermitting; then quick, and then ſlow again; in about ten minutes more, he grew red in the face, broke out into a ſweat, and his eyes opened; he continued in this ſweat, with rheum diſtilling from his mouth, about thirty minutes more; his reſpiration had been laborious, it now became freer; and at the expiration of ahout fifty minutes from his taking the draught, he roſe from his chair, by aſſiſtance, ſtaggering ſomewhat indeed, but he ſoon found the uſe of his legs, walked about the room, gazing like one in amazement.

I then, after ſome minutes, took him from the parlour where he had the fit, and walked an hour with him up ſtairs, in his drawing room; during this time he had ſpoke little more than aſking where he was, and ſome few queſtions beſides. Now I left him about two o'clock, and at three he walked down ſtairs, placed himſelf naturally at table, and cut up a neck of mutton, talked to his friend who dined with him, and made a tolerable meal.

I ſaw him in the evening. I found him drowſy, ſlobbering, and with his head and body hanging over an elbow chair; he went to bed early, but had no ſleep. He had had a ſmall ſtool in the morning.

16. I ſaw him early the next morning; found him much in the ſame poſture, his head drooping, ſlobbering, and his body hanging over the chair. I placed him on a ſopha, and bolſtered him up; thus he lay all day, and would eat nothing; he had an oily clyſter at night, and went to bed very early; fell faſt aſleep very ſoon, became very hot, red in the face, broke out into a profuſe ſweat, with free reſpiration, pulſe full 90.

17. Saw him early in the morning; found him in a profuſe ſweat and profound ſleep, with ſcarlet face, and pulſe full at 85. He lay in the very identical poſture he had taken when firſt put to bed, not a finger varying. Thus he lay the whole day, ſleeping and ſweating.

The bed was clean, and a terebinthinate clyſter was adminiſtered at night.

18. I ſaw him about eight o'clock in the morning; ſhook him, and he awaked; he immediately called for a baſin of ſage tea, and dry toaſt, which he eat and drank, from his own hand; the colour of his face was gone, but not altogether; he was weak, emaciated, and chop-fallen; his breath was ſhort, and ſome wheezing attended; his pulſe weak, ſmall, about 84. His friends were apprehenſive for him, and deſired Dr. Warren might ſee him; accordingly the Doctor ſaw him about noon this day. The clyſter had operated in the night, and the bed was full of the fouleſt offenſive evacuation. He was taken up, and cleaned, and during that operation had another moſt fœtid ſtool, as he was ſtanding, and nearly fainting at the time. He was now put upon a ſopha, where he was ſupported by bolſters, in a tolerably erect poſture, when the Doctor ſaw him. He was ordered a bliſter between the ſhoulders. He eat but little this day; had ſome ſleep on the ſopha, and more during the night; a clyſter was given at night; he ſpoke little, but ſeemed rational.

19. The bliſter roſe well, and he had a ſtool from the clyſter; pulſe 90. Dr. Warren now thought that as the Camphor had worked a change in him, it might be ſafe to continue it in a doſe of gr. x. bis die; accordingly it was ſo preſcribed.

The

The patient took nourifhment this day. His breath continued fhort, but the wheezing was gone off.

20. Had paffed a reftlefs night; had fweated much, and fouled his bed. Dr. Warren faw him again this morning, and took his leave.

22. The Camphor draughts continued to keep up perfpiration.

24. Was gaining ftrength and flefh, and was more converfable; played at cards, and franked his covers; appetite improved.

Nov. 3. He now had got confiderable ftrength, walked a mile moft days, and had never been helped by his fervant at meals fince the 15th of October. His bed indeed was generally wet, but feldomer foul.

Dr. Warren faw him this day, and thought him fit and ftrong enough to undertake a journey; which had been determined upon by his relations, who now thought his own manfion in the country the propereft place, to re-eftablifh his health.

Accordingly, Nov. 5, he fet out in a poft-chaife, knowing that he was going to his own houfe; he was chearful, rational, and minutely defcriptive of all the places he paffed through; he eat and drank with appetite, and continued well well throughout the day ; fome variation, however, was obferved in him after dinner, with figns of alienation of mind, and in this fituation he continued, now and then having fome gloomy intervals, but upon the whole greatly improved. His excretions were under his command through the whole journey, and continued fo afterwards ; and excepting one melancholy day, he was rational, and the natural man, till after his meal of dinner, when flight, but tranfient alienations of mind were vifible, and fo he continued till the 14th, when I loft fight of him.

It is obfervable in this cafe, that both in the years 1781 and 1783, upon the exhibition of Camphor in the dofe of ℈ji each time, a change was worked on the fenforium commune, and that the rational faculties each time returned.

I cannot help lamenting my not having had an opportunity of repeating this medicine, in the fame dofe of ℈ji ; as I think there is good foundation to fuppofe, that what had effected fo confiderable a change, might by proper repetition, (the patient's ftrength admitting of it) render that change permanent. But I would not in this cafe venture to prefcribe fuch a repetition, without being an eye witnefs of its effects, fects, and conducting him through the conse-quences. This opportunity I was robbed of, by the weak ſtate my patient was in, when ta-ken from me, at the expiration of ſix months.

This caſe likewiſe affords another proof of the impropriety of carrying patients to their own manſions, which are never favou-rable to the re-eſtabliſhing their intellects, by as much, as this my patient, who was in a high ſituation of life, and whoſe important con-cerns and worldly intereſts ruſhed in upon him, the moment he ſat down upon his own very conſiderable eſtate; the whole, and moſt minute circumſtances of which, he turned in his mind, in a moſt maſterly manner, he being himſelf a man of much genius, as well as knowledge; and when once the minds of ſuch men have been overſet, ſubjects, and objects of impor-tance, are the moſt likely to overthrow them again.

A moſt unfavourable circumſtance attended this gentleman's removal, which was to an old melancholy manſion, where a near relation had been confined for a number of years as inſane, and who finiſhed her days there.

The notorious change that happened ſo fre-quently after dinner induced me to give orders that he never might make a regular meal, but that he might be ſupplied with nouriſhment at proper intervals, without ſetting down to make a full meal; but this was neglected, and I think added to the diſadvantages he felt after I left him. Since the 14th of November I have no direct knowledge of his management, but I learn he is at length tranſported from his own houſe, and confined.

It often happens, that a concurrence of fortunate circumſtances promotes our ſucceſs in the cure of many diſeaſes; but here, a concurrence of unfortunate circumſtances, has ſeemingly contributed to our want of ſucceſs.

*Upper Charlotte Street,*

*Rathbone Place,*

March 29th, 1785.

